# Heterogenous cancer-associated fibroblasts related tumor microenvironment marked by CD10/KLF4/TIAM1 were identified in pancreatic adenocarcinoma by integrated transcriptomics

**DOI:** 10.3389/fimmu.2025.1557698

**Published:** 2025-04-14

**Authors:** Yu Wan, Qiong Hu, Kai Sun, Jing Shi, Limei Liu, Xiangsong Zhang, Jianjun Huang, Chulan Gong, Jinting Liu, Haijiu Wang, Jun Yan

**Affiliations:** ^1^ Department of Hepatobiliary and Pancreatic Surgery, Qinghai University Affiliated Hospital, Xining, China; ^2^ Department of Hepatobiliary Pancreatic Disease, Beijing Tsinghua Changgung Hospital, School of Clinical Medicine, Tsinghua Medicine, Tsinghua University, Beijing, China; ^3^ Department of Breast Surgery, The Affiliated Hospital of Guizhou Medical University, Guiyang, China; ^4^ Department of Cadre Medical, Guizhou Provincial People’s Hospital, Guiyang, China; ^5^ School of Biomedical Engineering, Tsinghua University, Beijing, China

**Keywords:** pancreatic adenocarcinoma, tumor microenvironment, spatial transcriptomics, cancer-associated fibroblasts, ScRNA-seq

## Abstract

Pancreatic ductal adenocarcinoma (PDAC) is a highly aggressive malignancy characterized by a dense and heterogeneous tumor microenvironment (TME) composed of various cancer-associated fibroblasts (CAFs). In this study, we explored the composition and proportions of CAF subtypes within the PDAC TME and identified three distinct CAF-related TME subtypes: iCAF-rich, myCAF-rich, and PSC-rich. We observed significant heterogeneity in CAF populations across different patients, which correlated with patient prognosis and the mechanical and fibrotic properties of the TME. Our analysis revealed that these CAF subtypes exhibit distinct gene expression profiles, with the myCAF-rich subtype showing upregulation of hypoxia- and glycolysis-related genes, such as LDHA. Furthermore, gene set and survival analyses demonstrated that specific CAF subtypes harbor unique protective and risk factors, which were non-overlapping between the subtypes. These findings suggest that the heterogeneity of CAF subtypes plays a critical role in PDAC progression and therapeutic response. By utilizing multiplex immunohistochemistry and spatial transcriptomics, we also identified key CAF subpopulations, such as iCAF_17, iCAF_19, and myCAF_12, which were found to interact closely with tumor cells and macrophages. In chemotherapy-treated patients, myCAFs were positioned at the tumor boundary, potentially acting as a barrier to tumor invasion. This study provides novel insights into CAF-related TME subtypes, offering a foundation for future therapeutic strategies targeting CAFs in PDAC.

## Introduction

Pancreatic ductal adenocarcinoma (PDAC) is recognized as an inherently aggressive malignancy with a dismal prognosis (1). At the time of diagnosis, only about 15% of patients present with tumors that are amenable to surgical resection, while the majority of cases are diagnosed at later stages, either with locally advanced or metastatic disease. Notably, approximately 50% of patients already have metastatic disease at the time of diagnosis, and over 80% of these metastases are confined to the liver, underscoring the highly metastatic nature of PDAC. This rapid progression and propensity for early dissemination significantly contribute to the difficulty in managing the disease. Despite advances in medical research, surgery remains the only potentially curative option for PDAC, although its application is limited to those with resectable tumors.

Unlike other gastrointestinal cancers, PDAC has not yet been fully classified into distinct subtypes with clear prognostic implications, which complicates the development of personalized treatment strategies (2). As a result, nearly all clinical guidelines recommend chemotherapy-based regimens as the standard first-line treatment for PDAC, often involving multi-agent chemotherapeutic approaches while other immunotherapy exhibited poor response (3). However, these treatments, while somewhat effective in some patients, are typically palliative rather than curative, with limited impact on overall survival. This highlights the urgent need for more precise biomarkers and novel therapeutic strategies to better target the underlying molecular mechanisms driving this lethal disease (4).

Fibroblasts, predominantly of mesenchymal origin, are ubiquitous components of the tumor microenvironment (TME), playing versatile and essential roles. They are critical for maintaining tissue homeostasis, regulating the proliferation and differentiation of adjacent epithelial cells, and orchestrating wound healing processes (4–6). Under the influence of malignant epithelial cells, resident fibroblasts undergo a phenotypic transformation into cancer-associated fibroblasts (CAFs), a process driven by abnormal wound healing stimuli. These transformed CAFs are increasingly recognized as pivotal players in oncogenesis, with their influence extending across a wide array of biological processes within the TME. Notably, CAFs are characterized by their functional heterogeneity, and their roles in cancer progression are far from unidirectional. Unlike many other types of tumors, PDAC is characterized by a uniquely dense stromal component, within which CAFs constitute a significant proportion (7). These CAFs not only contribute to the desmoplastic reaction but also play an active role in modulating the tumor microenvironment to favor tumor progression, metastasis, and therapeutic resistance (8). Given their abundance and multifaceted functions, CAFs have long been regarded as one of the most promising potential therapeutic targets in PDAC. Their ability to interact dynamically with both tumor cells and immune components makes them a critical focus for research aimed at improving PDAC treatment strategies. However, CAFs in PDAC are classically categorized into several subtypes, including iCAFs, myCAFs, and apCAFs (7). Emerging evidence suggests that targeting any single CAF subtype alone may not be sufficient to effectively suppress PDAC progression (8–10). This complexity highlights the critical importance of understanding the heterogeneity of CAFs, as well as the distinct roles and proportions of each subtype within the tumor microenvironment. While certain CAF subtypes actively promote tumor growth, invasion, and metastasis, others have been shown to exert tumor-suppressive effects (11). This functional diversity has led to conflicting results in preclinical and clinical studies, reflecting the duality of CAF functions in shaping tumor biology. Such inconsistencies have long perplexed researchers, prompting the hypothesis that the observed effects are not solely attributable to differences in fibroblast cell types, but rather to the complex composition and interplay of distinct CAF subpopulations within the TME (12). This nuanced understanding underscores the necessity of unraveling the specific contributions of CAF subtypes to TME remodeling, which may hold the key to resolving these longstanding ambiguities and advancing therapeutic strategies.

Recent studies have demonstrated that targeting CAFs, particularly through modulating their autophagic activity, could enhance the efficacy of conventional treatments. In preclinical mouse models, inhibition of CAF autophagy was shown to significantly increase the sensitivity of PDAC tumors to gemcitabine, a standard chemotherapy agent. This suggests that CAF-targeted therapies, either alone or in combination with chemotherapy, may offer a novel approach to overcoming chemoresistance and improving treatment outcomes for PDAC patients (13).

Here, we applied single-cell and spatial RNA-seq methods, based on the previous proposed CAFs subtypes, the CAF-related stratification of the PDAC TME is presented. And the key CAFs subtypes were identified to limit or promote the tumor invasion. Moreover, CD10 (*MME*) positive myCAFs were found as a novel tumor suppressed CAF subclusters, while *KLF4* positive and *TIAM1* positive iCAFs were considered as tumor promoted CAF subclusters. Taken together, it is promising to target these CAFs subtypes to overcome the difficulty in PDAC treatment.

## Materials and methods

### Ethics statement

This study enrolled 3 PDAC patients in Beijing Tsinghua Changgung Hospital. The diagnoses were confirmed by histopathology. This study was approved by the Institutional Ethics Committee of Beijing Tsinghua Changgung Hospital (Ethic code: 23355-0-01) in accordance with the ethical guidelines of the Institutional Ethics Committee and with the 1964 Declaration of Helsinki and its later amendments or comparable ethical standards. Written informed consent form was signed and obtained from the participant.

### Single-cell RNA-seq data processing

The raw data as downloaded from public database. The raw expression matrix from each dataset was imported into R software to filter out low-quality cells. The criterion is that less than 200 genes/cell, 200 UMIs/cell or more than 40% mitochondria genes cells were dropped. The analysis tool was ‘Seurat’ R package (v5.1.0). Batch effect was corrected similar to published studies (14). The Harmony algorithm (harmony package v0.1.1) was applied to correct batch effects for each dataset, which was a widely used, sensitive and accurate integration of single-cell data algorithm (15).

Next, gene expression levels were normalized and scaled following the standard pipeline. A total of 2, 000 highly variable genes were used to conduct PCA reduction dimension. Harmony R package was used with the top 20 PCs to correct the potential batch effect. For primary analyses, unsupervised cell clusters were acquired by graph-based clustering approach (The top 20 dims were selected), and visualized by UMAP dimensionality reduction. STARTRAC-dist indices were calculated via formula: Ro/e = observed/expected, in which Ro/e is ratio of observed cell number over the expected cell number of a given combination of cell type or tissue. The method was developed and validated in previous studies (16).

### Spatial RNA-seq data processing

The expression data and tissue section images were obtained from previous dataset (17). Raw expression matrix from each sample was imported into R merged using the ‘Seurat’ R package (v5.1.0), or Python (v3.10) for downstream analysis. Next, gene expression levels were normalized and scaled to all genes. For primary analyses, unsupervised niches were acquired by graph-based clustering approach (The top 20 dims were selected), and visualized by UMAP dimensionality reduction and spatial distribution. The niches were subsequently annotated according to the gene expression and cell composition. Raw scRNA-seq matrix with annotation of cell types was used as reference for stRNA-seq deconvolution. Cell2location (v0.1.3), a principled Bayesian based deep-learning model, was applied to estimate cell abundance and types in spatial distribution (18). Cell2location was run with default parameters, with the exception of cells_per_spot which was set to 60. Each Visium section was analyzed separately. The estimated abundance for each cell type was visualized following the cell2location tutorial. The convergence of results was inspected after 20,000 epochs iteration.

### Cell-cell communication

For single-cell RNA-seq data, Cellchat R package (v2.1.2) was applied to analyze cell-cell communication based on ligands and receptors inferred, implemented with the normalized expression matrix with full database and default parameters (19).

### Functional enrichment analysis

The clusterProfiler R package (v4.10.1) was used to conduct enrichment analyses (20). The reference gene sets were selected from Gene Ontology (GO) gene sets (C5), Kyoto Encyclopedia of Genes and Genomes (KEGG) Medicus gene sets (CP: KEGG_MEDICUS) and hallmark gene sets (H) databases (https://www.gsea-msigdb.org/). The other gene set-based enrichment analysis was carried out with GSVA R package (21). The statistic threshold was set to p-value less than 0.05 without individual statement.

### Immunohistochemistry

PDAC tissues underwent mIHC staining of CK19, αSMA, CD68, TIAM1, KLF4, and CD10. Standard operating procedures were employed for IHC, mIHC, and HE staining. The visualization of IHC staining was carried out using Akoya Biosciences The PhenoImager HT 2.0. Beijing Histova Biotechnology was used for mIHC staining following the manufacturer’s instructions. The primary antibodies used for mIHC staining were as follows: anti-CK19 (1:200 dilution, ZM-0074, ZSG-Bio, China), anti-CD68 (1:200 dilution, OTI8A4, OriGene, China), anti-αSMA (1:200 dilution, AF1032, Affinity Biosciences, China), anti-CD10(1:200 dilution, DF7446, Affinity Biosciences, China), anti-KLF4 (1:200 dilution, AF2737, Affinity Biosciences, China), anti-TIAM1 (1:200 dilution, TA382521, OriGene, China). Results of mIHC staining were evaluated using HALO software (V.3.5.3577), Indica Labs, Albuquerque, USA).

### Statistical analysis

Statistical analyses were conducted by R software (v4.3.1). Two-tailed unpaired student’s t-test was utilized to show the significant difference between groups. A p-value of < 0.05 was considered to indicate a statistically significant result. Significant differences were noted as * p-value < 0.05, ** p-value < 0.01, and *** p-value < 0.001. Results were visualized using the ggplot2 R package (v3.5.1).

## Results

### Heterogenous infiltrated CAFs subtypes in PDAC TME

**Figure 1:**
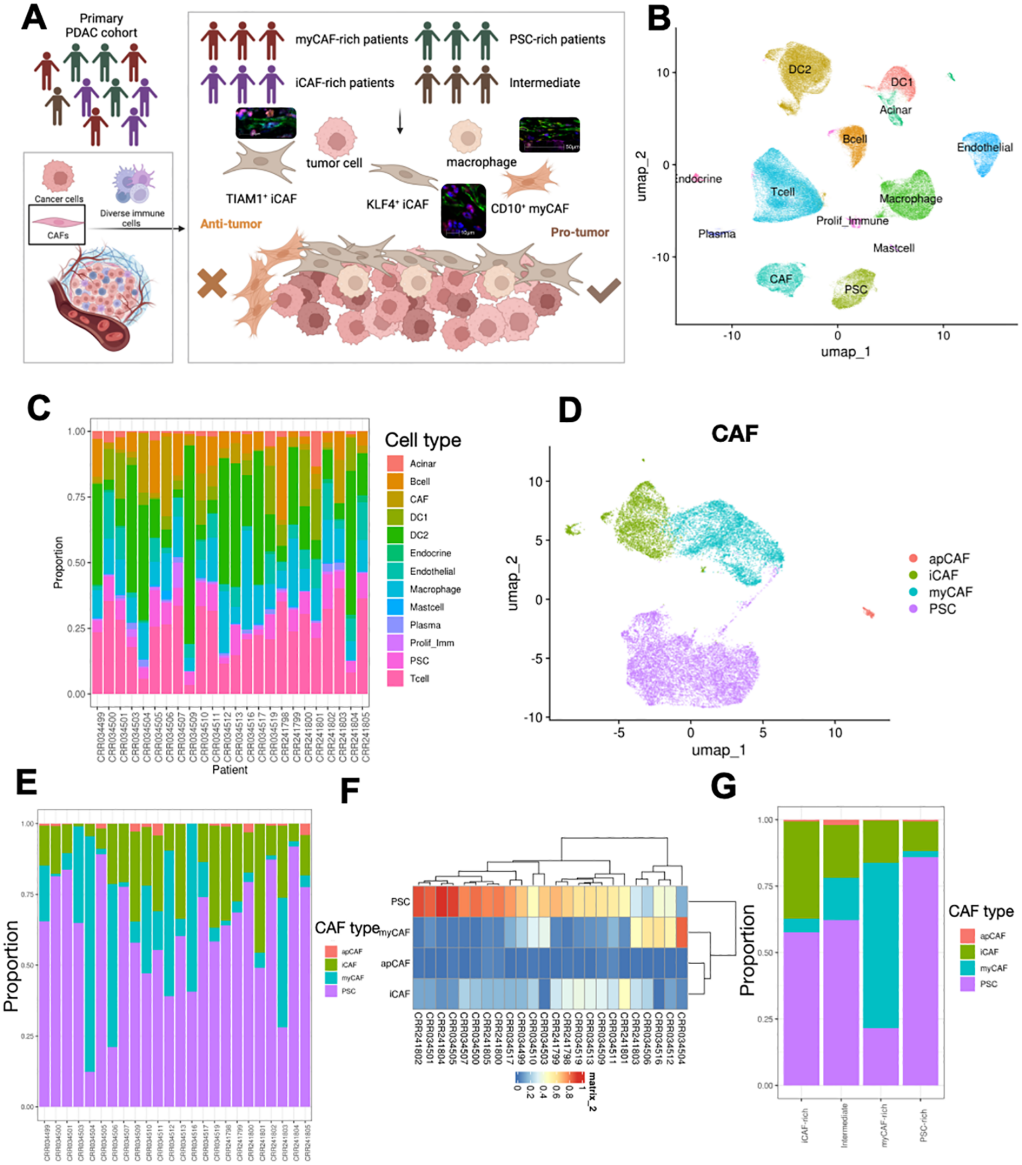
Overall design of experiment and alignment of scRNA-seq and stRNA-seq. **(A)** Schematic showing workflow, and sampling methods of PDAC samples. **(B)** UMAP visualization of the cell types in PDAC TME. **(C)** Proportion of different cell types in PDAC samples. **(D)** UMAP visualization of CAFs and PSCs subset from integrated dataset. **(E, F)** Proportion of different subtypes of PSCs and CAFs in each patient, showed in bar **(E)** and clustered heatmap **(F)**. **(G)** Four CAF-related TME subtypes were identified, and the proportions of CAFs and PSCs in each subtype were calculated.

The tumor microenvironment (TME) of pancreatic ductal adenocarcinoma (PDAC) is characterized by significant heterogeneity. To investigate this feature, we designed a research workflow that included single-cell RNA sequencing (scRNA-seq) data from n=24 treatment-naive patients with primary PDAC, as well as samples from n=2 PDAC patients, which consisted of cases that received and did not receive chemotherapy, subjected to Spatial RNA sequencing (stRNA-seq) (**Figure 1A**). Initially, we obtained the scRNA-seq results for PDAC, and after implementing stringent data quality control measures, we identified 145,434 high-quality cells for subsequent analysis. Utilizing cell marker information from previously published studies, we performed cell annotation, resulting in a high-quality single-cell atlas of the PDAC TME (**Figure 1B**). The major cell subpopulations include T cells, macrophages, ductal cells, and fibroblasts. In the tumor microenvironment of different pancreatic cancer patients, various cell components exhibit notable heterogenous characteristics (**Figure 1C**). PDAC is characterized by a tumor microenvironment predominantly composed of fibroblast components. In this microenvironment, the proportion of tumor cells is relatively low compared to other tumor types, which leads us to focus on the fibroblast components. The fibroblast components in the pancreatic cancer microenvironment consist primarily of cancer-associated fibroblasts (CAFs) and pancreatic stellate cells (PSCs). We extracted these fibroblast components and performed a re-clustering and classification (**Figure 1D**). Consistent with previous studies, we defined three types of tumor-associated fibroblasts: antigen-presenting activated fibroblasts (apCAF), myofibroblasts (myCAF), and inflammatory cancer-associated fibroblasts (iCAF). Next, we categorized the fibroblast components for each pancreatic cancer patient and discovered significant differences in the composition of these components within the tumor microenvironments of different patients. Some patients exhibited a higher proportion of apCAF, while others predominantly featured PSC (**Figure 1E**). This led us to question whether patients could be classified into distinct subtypes based on the heterogeneity of fibroblast components. To explore this possibility, we conducted a clustering analysis of the fibroblast composition in the tumor patients (**Figure 1F**). We found that a subset of patients primarily exhibited PSC components, while another group was dominated by myCAF, and some patients displayed iCAF as their main component. Consequently, we categorized these patients into three distinct subtypes: myCAF-rich, PSC-rich, and iCAF-rich. Given the minimal infiltration of apCAF, we did not define this group of patients separately. Instead, we categorized the remaining patients as intermediate type. Next, we conducted a re-integration analysis of various types of pancreatic cancer patients, focusing on the proportions of different fibroblast components infiltrating their tumor microenvironments. Consistent with our expectations, each patient type exhibited characteristic infiltrations of fibroblast components. However, contrary to our assumptions, some cell subpopulations appeared to show constitutive infiltration; for instance, patients classified as iCAF-rich exhibited increased iCAF infiltration, yet the highest proportion of infiltrating cells remained PSC (**Figure 1G**). This observation suggests that fibroblast components may possess further heterogeneity that influences the overall composition of the microenvironment.

### MyCAF-rich TME identified as typical CAF-rich TME with unfavorable outcomes

**Figure 2:**
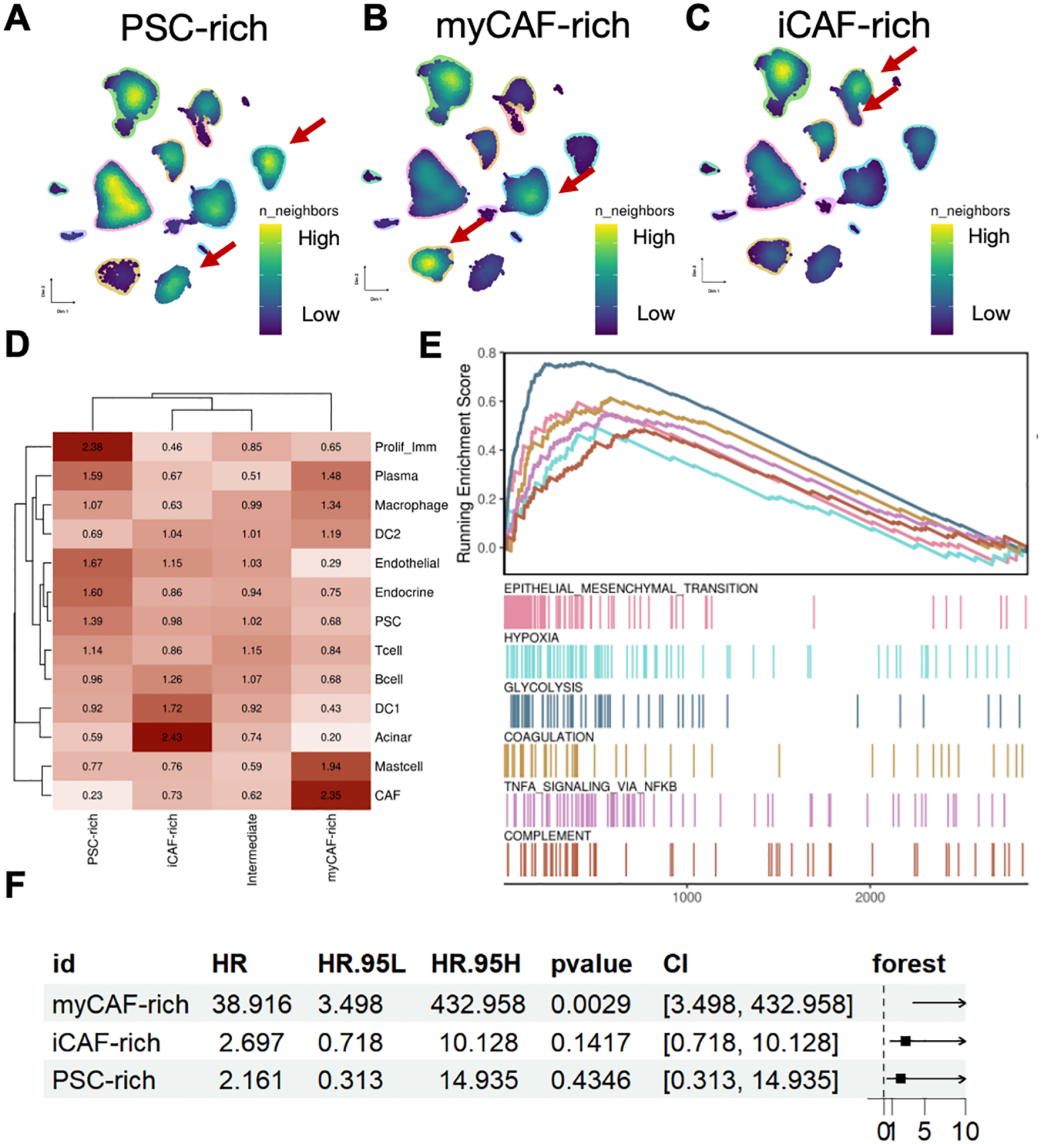
MyCAF-rich TME is associated with typical niche and unfavorable clinical outcome. **(A–C)** UMAP showed cell density in each TME subtypes. The red arrows indicated representative cell types. **(D)** The Ro/e value (log scaled) of each cell type in different TME subtypes. **(E)** Geneset enrichment analysis suggested the myCAF-rich TME were shown regulated EMT, hypoxia, and glycolysis activity. **(F)** Forest plot showed the unfavorable clinical outcome of myCAF-rich TME signatures, while the other iCAF-rich and PSC-rich were not significant.

To further investigate the differences in the microenvironments of pancreatic cancer patients across different subtypes, we presented the composition of cells and the richness of various cell subtypes in the tumor microenvironments of three distinct patient groups (**Figures 2A–C**). We observed significant differences in cell density between patients with different fibroblast types. In the myCAF-rich patient subgroup, we found that this group contained a higher abundance of cells traditionally annotated as CAF components (**Figure 2D**). Consequently, we conclude that these patients represent a true aggregation of fibroblast components within pancreatic cancer. Meanwhile, we found that CAFs, acinar cells, and proliferating immune cells were enriched in three patient groups. The majority of cells in patients classified as intermediate were found as intermediate abundance between the different subpopulations, providing further evidence for our definition of this intermediate group. Next, we were curious about the expression characteristics of the TME in this group of fibroblast-enriched patients. In the myCAF-rich subgroup, we observed upregulation of pathways related to glycolysis, hypoxia, and epithelial-mesenchymal transition, all of which are associated with tumor onset and progression and represent characteristic pro-cancer pathways in pancreatic cancer. Additionally, we found upregulation of several inflammation-related pathways, such as tumor necrosis factor alpha (TNF-α) and the complement pathway (**Figure 2E**).These pathways were considered as potential regulator of immunotherapy (22, 23). However, for the other two subgroups, no obvious pathways were enriched related to neogenesis and other biological process (**Supplementary Figure 2**). Subsequently, we sought to determine whether the expression profiles of these patients influenced their disease survival prognosis. We incorporated external data (TCGA cohort) for transcriptomic analysis and statistically analyzed the characteristic genes of the three patient subtypes. Our results indicated that only the expression profile of myCAF-rich patients was significantly associated with poor prognosis, whereas the expression profiles of the other two groups did not appear to serve as survival predictors (**Figure 2F**). Thus, we conclude that these patients represent those traditionally considered to have an accumulation of fibroblast components within the pancreatic cancer microenvironment, characterized by hypoxia and glycolysis, and that their expression profiles can predict poor prognosis in PDAC.

### Different CAFs-related TME subtypes have distinct hazard and protect signatures

**Figure 3:**
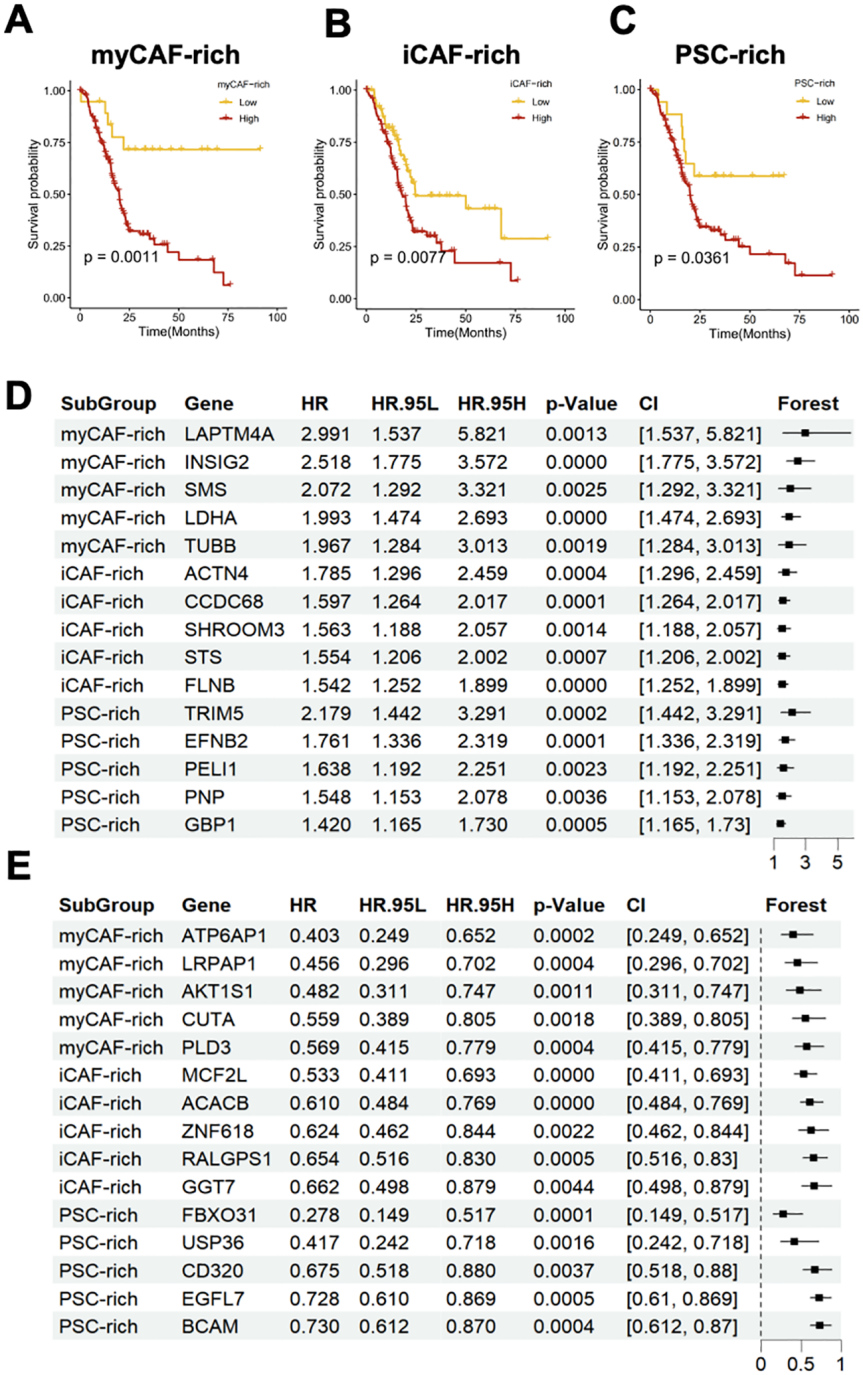
Different CAFs-related TME subtypes have heterogenous clinical outcomes and distinct feature genes. **(A–C)** Survival analysis indicated that the myCAF-rich, iCAF-rich, and PSC-rich TME subtypes have distinct clinical outcomes. The myCAF-rich TME was considered as the most significant classifier for overall survival **(D, E)** Forest plot showed the top 5 hazard **(D)** and protect **(F)** genes and their effect for predicting clinical outcomes.

To further investigate the relationship between different CAF subtypes in the tumor microenvironment and the prognosis of PDAC patients, we introduced an external cohort to assess the predictive ability of marker genes for three distinct tumor microenvironment subtypes (**Figures 3A–C**). We found that the marker genes of all three subtypes were able to predict poor prognosis in the external cohort. However, in the myCAF-rich tumor microenvironment, this gene set showed the strongest association with adverse prognosis, which may be related to our hypothesis that this subtype represents the classic PDAC TME. Given the strong heterogeneity observed among different tumor microenvironment subtypes, we aimed to identify the specific genes that serve as major prognostic variables. For the three distinct tumor microenvironment subtypes, we performed Cox regression analysis to identify the top five marker genes with the most significant protective or adverse prognostic effects in each subtype. In the myCAF-rich subtype, we identified classic poor prognosis genes, such as LDHA, further reinforcing the notion that this subtype represents a more common tumor microenvironment (**Figures 3D, E**). Additionally, we discovered several prognostic marker genes in the iCAF and PSC-rich subtypes, including ACTN4, CCDC68, TRIM5, EFNB2, MCF2L, ACACB, FBXO31, and USP36. The discovery of these marker genes highlights the substantial heterogeneity within different tumor microenvironment types. Furthermore, it suggests that subclassifying PDAC patients based on CAF-related TME subtypes and adopting a precision treatment approach may be a promising strategy to enhance therapeutic outcomes in the future.

### PDAC TME remodeled by iCAF subtypes by interacting with tumor cells and immune cells

**Figure 4:**
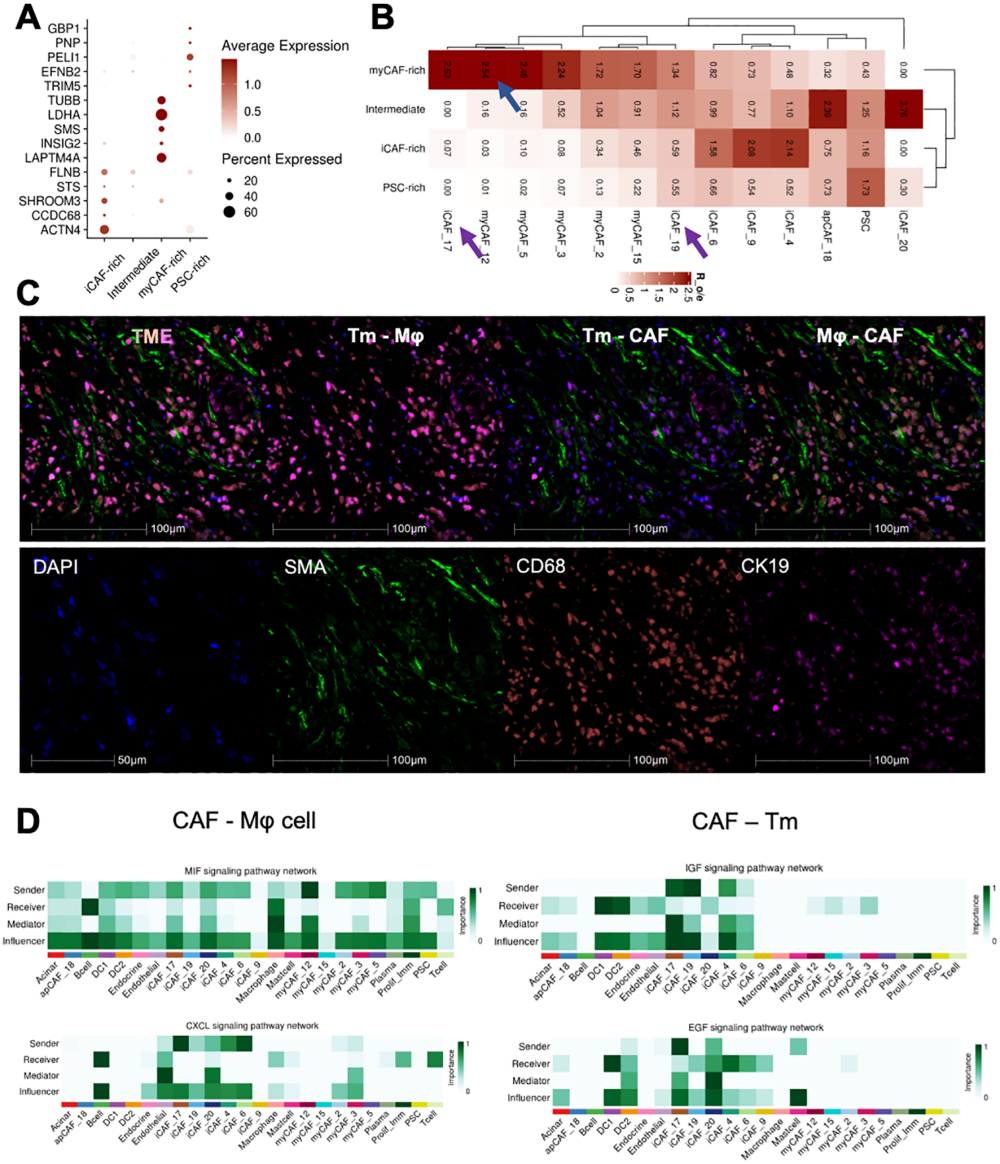
Different CAFs-related subtypes TME cross talked with tumor cells and immune cells. **(A)** Dotplot exhibited the expression of clinical prediction signatures in distinct CAFs-related subtypes patients. **(B)** The CAFs and PSC subtypes infiltration ratio in four subtypes TME. **(C)** Multi-color IHC staining showed the tight co-localization of CAF, Macrophage(Mφ), and tumor cells(Tm). **(D)** Ductal cells and Macrophage interacted with iCAF_17 and iCAF_20 by IGF, EGF and MIF, CXCL signaling.

PDAC tumors exhibit a high degree of heterogeneity in their associated cellular components. To further investigate the impact of CAFs on the tumor microenvironment, we extracted CAFs and PSC cells, which are developmentally related. Using classic markers reported in the literature, we classified these cells into 21 subgroups and visualized their distribution using UMAP dimensionality reduction (**Supplementary Figures 2A, B**). Despite above findings showing that various CAF subgroups infiltrate different patient cohorts, this infiltration appears to be non-specific. However, we discovered that CAF-related marker genes are specific to the CAF cell subtype in each patient (**Figure 4A**). While CAFs are generally present in different TME subtypes, specific tumor microenvironments tend to express genes that are characteristic of the corresponding CAF subtypes. This observation helps explain why diverse cellular components coexist in the tumor microenvironment, yet exhibit distinct prognostic and biological behaviors. The decisive role of key genes in certain tumor-associated fibroblast subgroups may be a contributing factor (**Figure 4B**).

To investigate how these key subgroups impact the tumor microenvironment, we conducted intercellular interaction analyses across all CAF subgroups and other cellular components. (**Supplementary Figure 2C**). Furthermore, mIHC staining was applied to evaluate the co-localization of CAF(SMA), macrophage (CD68), and malignant tumor cells (CK19), which was confirmed tightly adjunct with each other (**Figure 4C**). We found that the iCAF_17 and iCAF_19 groups were the most active in receiving and emitting signals (**Figure 4D**). Further detailed analysis of their signaling pathways revealed that these groups influence both tumor cell growth and T-cell chemotaxis. Additionally, we observed that myCAF_12 cells strongly interact with immune cells and regulate the tumor microenvironment via the MIF signaling pathway. In conclusion, we have identified three distinct CAF subgroups that collaboratively shape the PDAC tumor microenvironment by modulating both tumor cells and immune cells.

### CD10+ myCAF inhibit while KLF4+/TIAM1+ iCAF promote tumor invasion in chemotherapy

**Figure 5:**
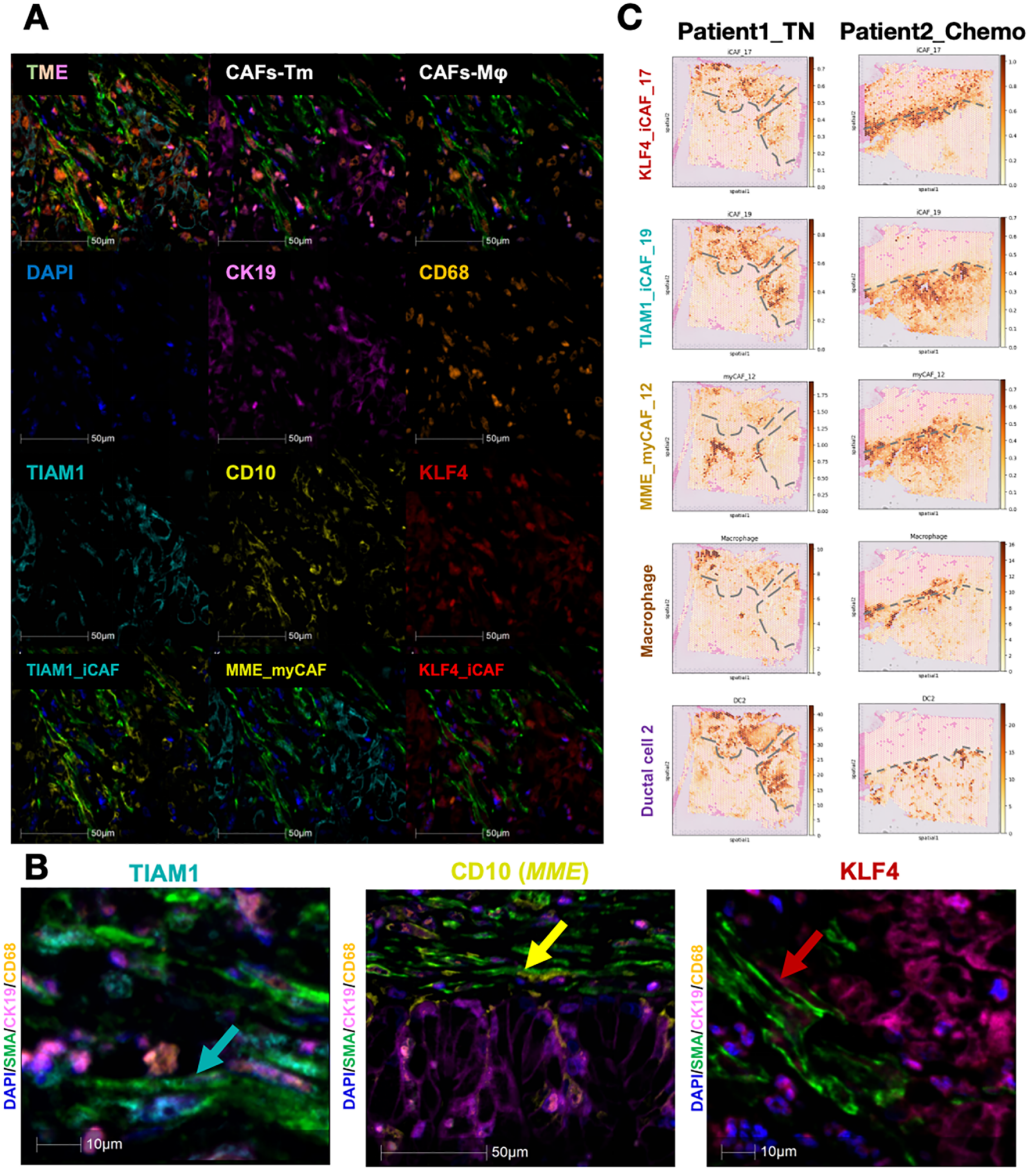
Existence and spatial arrangement of three key subtypes CAFs. **(A, B)** Multi-color IHC staining exhibited the existence of each CAFs subtypes. CD10(*MME*) for myCAF_12, KALF4 for iCAF_17, and TIAM1 for iCAF_19 **(C)** The spatial arrangement of major cell types and the dynamic change before and after chemotherapy. The grey dashed lines showed the boundary of tumor.

To verify the actual presence of these fibroblast subgroups, we stained tissue sections from PDAC patients. First, we identified the top DEGs as representative gene marker of three CAFs subclusters: CD10(*MME*) for myCAF_12, *KLF4* for iCAF_17, and *TIAM1* for iCAF_19. The specific of genes was evaluated in all CAFs (**Supplementary Figure 3A**). Using SMA and subgroup-specific marker genes, we identified the presence and spatial localization of the three CAF subgroups in the PDAC tumor microenvironment (**Figure 5A**). Simultaneously, tumor cells were mapped within the same sections, revealing that different CAF subgroups have characteristic spatial distributions relative to tumor boundaries (**Figure 5B**).

Since CAFs influence the extracellular matrix and collagen components in the tumor microenvironment, they are pivotal in modulating the therapeutic response in pancreatic cancer. As chemotherapy remains the standard treatment for PDAC, we explored whether CAFs affect neoadjuvant chemotherapy outcomes. We analyzed two PDAC patients, one untreated and the other treated with chemotherapy, to map the spatial distributions of tumor and adjacent cells (**Figure 5C**). The borderline of tumor area was determined by their H&E staining as well as duct_2 cell distribution. In the untreated patient, iCAFs were predominantly infiltrating tumor regions, while myCAFs were confined to non-tumor areas. In contrast, in the treated patient, both iCAFs and myCAFs localized to the tumor boundary, suggesting that myCAFs may act as a physical barrier to tumor progression. This observation aligns with previous findings that iCAFs are tumor-promoting, while myCAFs exhibit tumor-suppressive effects. These findings were further corroborated by mIHC staining, showing that myCAFs are enriched at tumor boundaries, whereas iCAFs are more closely associated with intratumoral regions.

## Discussion

PDAC is an aggressive malignancy with a notoriously poor prognosis (1). The differential functions of these CAF subtypes in promoting tumor growth, metastasis, and immune evasion underscore the necessity of refining CAF classification and investigating their compositional dynamics. Such insights could provide valuable clues to deciphering their contributions to PDAC initiation, progression, and metastasis, ultimately aiding in the development of more precise and effective therapeutic strategies. In this study, we focused on the composition and proportions of CAFs in PDAC, uncovering substantial heterogeneity in CAF populations across different patients (**Figure 1**). Our findings revealed that CAF subtypes, including PSCs, exhibit distinctive distribution patterns among patients, suggesting a potential link between these patterns and patient prognosis (24, 25). This variability in CAF composition may play a pivotal role in shaping the unique fibrotic and mechanical properties of the PDAC tumor microenvironment (5, 26). Such properties are known to influence tumor progression, therapeutic response, and metastatic potential. These results underscore the need to further explore the relationship between CAF heterogeneity, tumor biomechanics, and patient outcomes to identify new strategies for personalized PDAC therapies (**Figures 1F, G**).

Heterogenous CAF-related TME subtypes were found related to biologic behaviors and prognosis in PDAC. Based on our findings, we classified PDAC TMEs into three distinct subtypes: iCAF-rich, myCAF-rich, and PSC-rich (**Figures 2A-C**). This classification revealed significant differences in the cellular subpopulations present within each TME subtype, encompassing both immune and stromal components (**Figure 2D**). These differences suggest that CAF-based TME subtyping reflects unique tumor immune microenvironments (TIMEs), which may have important implications for disease progression and therapeutic response in PDAC. The interplay between CAF composition and immune dynamics could potentially influence tumor growth, immune evasion, and sensitivity to treatments. Our results highlight the importance of understanding the CAF-driven heterogeneity of TMEs, which may provide a foundation for tailoring PDAC therapies to specific TME subtypes in the future. In the myCAF-rich TME, we observed upregulation of pathways associated with hypoxia and EMT, aligning with the classical perspective of PDAC TME characteristics (**Figure 2E**) (27, 28). These findings suggest that myCAF-rich TMEs retain a prototypical desmoplastic and aggressive tumor microenvironment (8). Furthermore, after identifying the marker genes for different CAF-related TME subtypes, we confirmed that the features of the myCAF subtype are strongly associated with PDAC prognosis (**Figure 2F**). This association highlights the critical role of myCAFs in driving tumor progression and underscores their potential as biomarkers for risk stratification and therapeutic targeting in PDAC. Further gene set and survival analyses revealed that distinct CAF subtypes harbor specific protective and risk factors, which are non-overlapping across the different CAF-related TME subtypes (**Figure 3**). These findings provide strong evidence for the existence of distinct CAF-related TME subtypes in different patients, emphasizing the heterogeneity of PDAC TMEs. Notably, in the myCAF-rich subtype, we identified the upregulation of hypoxia- and glycolysis-related genes, particularly LDHA, a key metabolic regulator (29). The hypoxia TME was widely acknowledged with drug resistance (30). This observation reinforces the association between the myCAF-rich TME and a metabolically reprogrammed, aggressive tumor environment, further linking it to adverse patient outcomes.

CAFs-related TME subtypes educated by different CAFs clusters, and distinct CAFs remodeling the TME with tumor cell (ductal 2 cells) and macrophages. Our analysis revealed that genes associated with TME classification were characteristically expressed within their corresponding tumor microenvironments. However, a closer examination of all identified CAF subpopulations highlighted notable differences, particularly in subtypes such as iCAF_17, iCAF_19, and myCAF_12 (**Figures 4A, B**). These specific CAF subpopulations exhibited significant variations across CAF-related TME subtypes and were identified as the most active “key minority” subpopulations within the TME (**Figures 4C, D**). Further investigation into their cellular communication networks showed that their primary interaction partners were macrophages and tumor cells, underscoring their central role in shaping the TME (31, 32). Immunohistochemical staining of the microenvironment provided additional evidence, demonstrating that these key CAF subpopulations and their interacting partners were prominently co-localized within tumor regions (**Figure 4D**). This spatial clustering highlights their potential importance in modulating tumor progression and immune responses.

Furthermore, the marker genes for these CAF subpopulations were defined as *TIAM1*, *MME* (CD10), and *KLF4* (**Supplementary Figure 3A**). These genes were found to be significantly upregulated and highly specific within their respective CAF subtypes. Using multiplex immunohistochemistry, we successfully localized these novel CAF subpopulations within the TME, revealing their close association with tumor cells and macrophages (**Figure 5**). This interaction was further validated through spatial transcriptomics, which corroborated the spatial positioning of these CAFs in relation to other cell types in the microenvironment.

Interestingly, the spatial distribution of these CAF subpopulations also appears to be associated with chemotherapy treatment (**Figure 5C**). In chemotherapy-treated patients, myCAFs seemed to form a barrier at the tumor boundary, potentially acting as a physical shield to limit tumor invasion, while iCAFs were observed to cluster at the tumor periphery (33). This observation suggests that these CAF subpopulations may play distinct roles in modulating tumor progression and chemoresistance (34). Overall, our study defines CAF-related TME subtypes and identifies three critical CAF subpopulations, providing novel insights into potential therapeutic strategies targeting CAFs in PDAC. These findings offer a promising direction for refining treatment approaches aimed at modifying the TME to improve patient outcomes.

## Code availability

This study did not generate original codes. All software and algorithms used in this study are publicly available and listed in the *Materials and methods* section. R and python codes used to analyze data and generate figures are available upon request.

## Data Availability

The original contributions presented in the study are included in the article/**Supplementary Material**. Further inquiries can be directed to the corresponding authors.

## Supplementary material

Supplementary Figure 1
**(A)** Quality control of scRNA-seq data. **(B)** Validation of potential batch effect of scRNA-seq data. The colors represented each PDAC sample. **(C)** The expression markers of each cell types in each cell cluster.

Supplementary Figure 2
**(A)** The expression markers of each CAF subtype in each cell cluster. **(B)** The distribution of CAF subtypes in UMAP reduction. Arrows indicated the location of significantly changes subtypes. **(C)** Heatmap profiled the signaling patterns from and target all cell types in the PDAC TME. ICAF_17 and iCAF_20 were identified as the most active receivers.

Supplementary Figure 3
**(A)** The expression markers of given three CAF subtypes of their marker genes MME, KLF4, and TIAM1. **(B)** Multi-color staining of PDAC patients to validate the existence of CAF subtypes.
