# Supplementary material for: Heterogenous cancer-associated fibroblasts related tumor microenvironment marked by CD10/KLF4/TIAM1 were identified in pancreatic adenocarcinoma by integrated transcriptomics
Source: Front Immunol. 2025 Apr 14;16:1557698. doi: 10.3389/fimmu.2025.1557698 (PMC12038268; doi:10.3389/fimmu.2025.1557698)
Supplement: Supplementary Figure 1 — (A) Quality control of scRNA-seq data. (B) Validation of potential batch effect of scRNA-seq data. The colors represented each PDAC sample. (C) The expression markers of each cell types in each cell cluster. [file DataSheet1.pdf]

Supplementary Figures

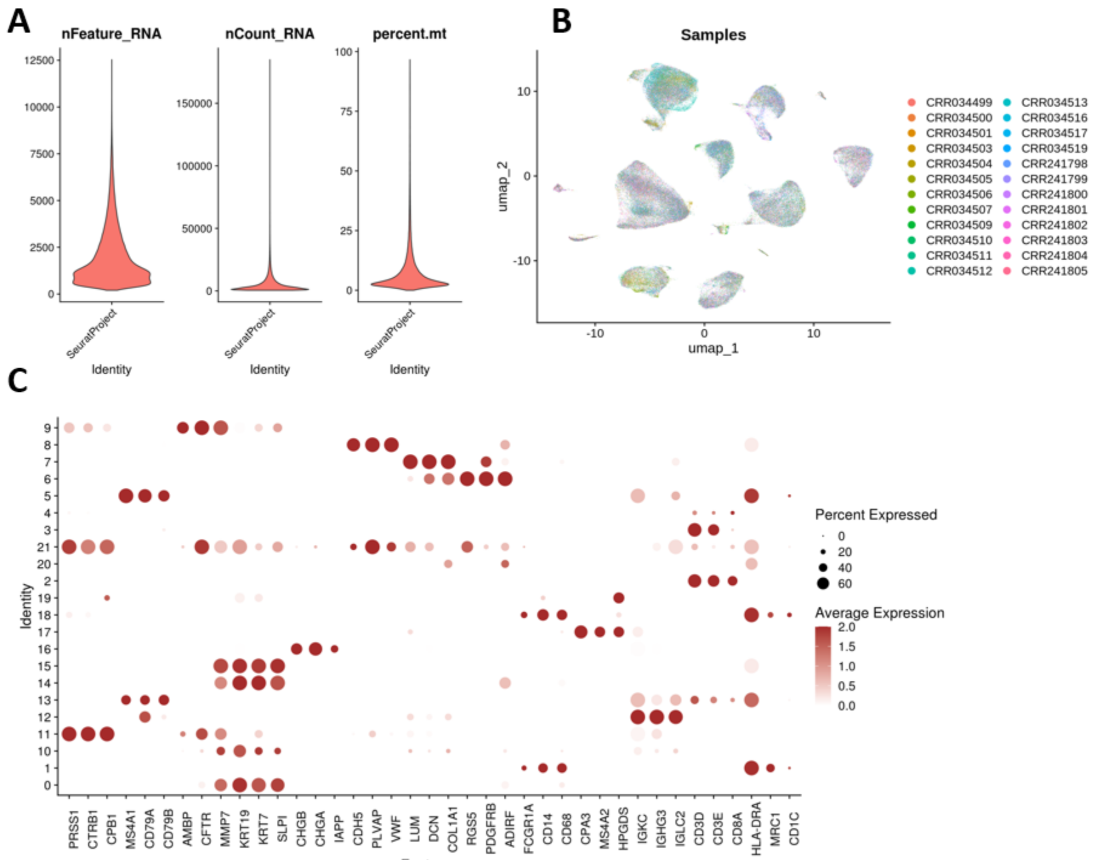

Supplementary Figure 1. (A) Quality control of scRNA-seq data. (B) Validation of potential batch effect of scRNA-seq data. The colors represented each PDAC sample. (C) The expression markers of each cell types in each cell cluster.

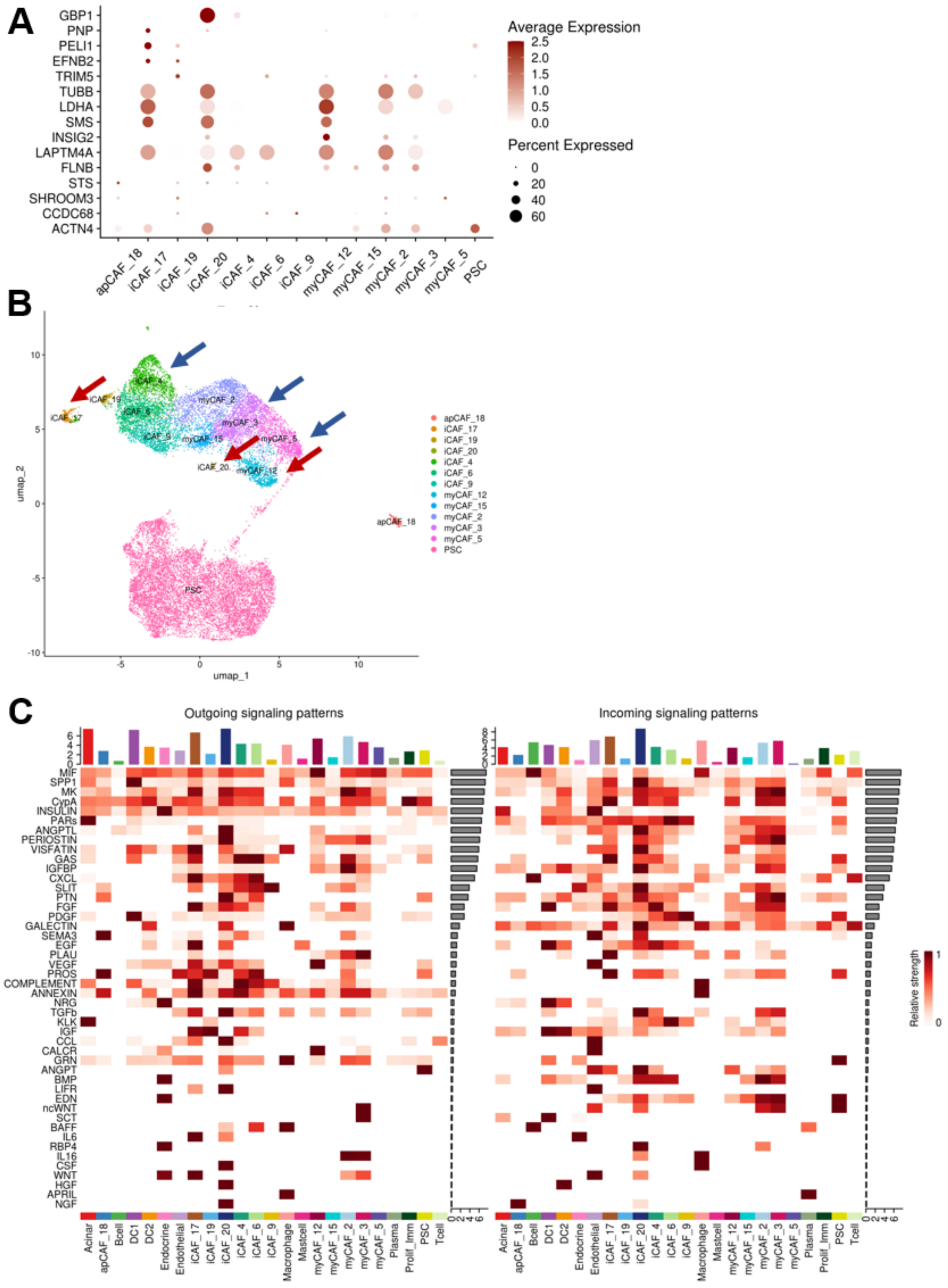

Supplementary Figure 2. (A) The expression markers of each CAF subtype in each cell cluster. (B) The distribution of CAF subtypes in UMAP reduction. Arrows indicated the location of significantly changes subtypes. (C) Heatmap profiled the signaling patterns from and target all cell types in the PDAC TME. ICAF_17 and iCAF_20 were identified as the most active receivers.

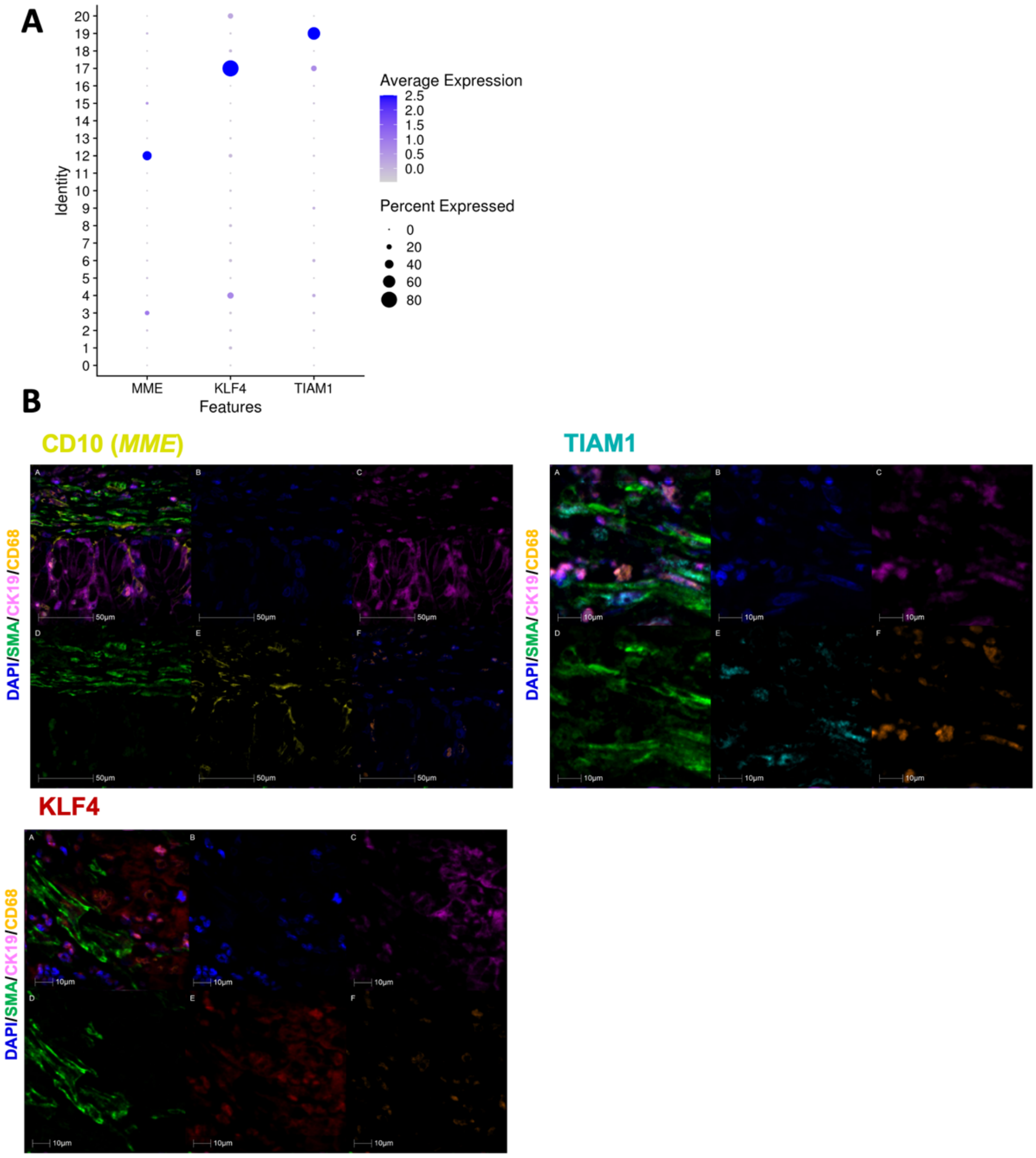

<u>**Supplementary Figure 3.** (A) The expression markers of given three CAF subtypes of their marker genes MME, KLF4, and TIAM1. (B) Multi-color staining of PDAC patients to validate the existence of CAF subtypes.</u>
